# Lessons learned in co‐creating a Virtual Village for people ageing with HIV

**DOI:** 10.1002/jia2.26084

**Published:** 2023-05-23

**Authors:** Jasmine L. Lopez, Andrea N. Polonijo, Annie L. Nguyen, Karah Y. Greene, Jerome T. Galea, Moka Yoo‐Jeong, Jeff Taylor, Brandon J. Brown

**Affiliations:** ^1^ Department of Psychology University of California Riverside California USA; ^2^ Department of Sociology and the Health Sciences Research Institute University of California Merced California USA; ^3^ Herbert Wertheim School of Public Health and Human Longevity Science University of California, San Diego La Jolla California USA; ^4^ College of Behavioral and Community Sciences, School of Social Work University of South Florida Tampa Florida USA; ^5^ Bouvé College of Health Sciences Northeastern University Boston Massachusetts USA; ^6^ HIV+Aging Research Project–Palm Springs (HARP‐PS) Palm Springs California USA; ^7^ Department of Social Medicine, Population and Public Health School of Medicine University of California Riverside California USA

**Keywords:** community, key and vulnerable populations, North America, public health, quality of life, structural interventions

1

The COVID‐19 pandemic emerged as a global threat in early 2020, resulting in new psychosocial stressors and widespread lifestyle changes. These stressors contributed to heightened depression, isolation, anxiety, and in some cases, exacerbation of post‐traumatic stress disorder [1]. Numerous studies have examined the impact of the COVID‐19 pandemic on mental health in the general population [2] but research addressing its impact on increased isolation among the growing number of older people living with HIV (OPLH) in the United States is limited [3]. Long‐term survivors have shared trauma stemming from profound losses in their social circle due to HIV/AIDS, which younger people living with HIV may not experience as a result of the development of therapeutics.

Virtual Villages are online spaces that create positive connections among older adults, while delivering interventions and supporting individuals who are “ageing in place”—that is, living in their community of choice during later life [4]. It is imperative for positive social activities to be easily accessible for OPLH due to their heightened prevalence of depression originating from past trauma [5], elevated rates of psychological distress and increased barriers to successful ageing faced while living through the COVID‐19 pandemic [6]. Previous research shows that interventions that address psychosocial barriers among OPLH are vital to promoting successful ageing [7, 8, 9]. As such, our objective was to construct a Virtual Village to support and connect OPLH and promote mental wellbeing through stress relief, knowledge and confidence building.

Online platform‐based socialization has been shown to strengthen emotional support systems, improve planning skills and enhance coping among people living with HIV [10]. We designed a Virtual Village to supply health, community‐based and personal resources for OPLH aged 50+, provide a safe space for participants to create support groups and attenuate the negative impacts of social isolation. Below, we discuss lessons learned during the co‐creation of this Virtual Village.

Our first step in creating the Virtual Village was to develop a strong partnership with a community advisory board (CAB) of 24 OPLH from three U.S. regions. With trust established and a clear mission for the Virtual Village, we constructed a list of 28 potential characteristics the Virtual Village pilot could offer. The CAB rank‐ordered the attributes and identified the five most important: free‐to‐use, chat function availability, registration and security measures, access to community resources and ability to make subcommunities.

Next, we compared and contrasted seven potential online platforms for hosting the Virtual Village and selected Discord based on its compatibility with desired attributes, and other important factors, including privacy mechanisms, accessibility and moderation features, function automation, video chat, direct messaging and technological support. We established a Discord server and adapted it in response to feedback from CAB members.

Twenty‐four OPLH, who were not CAB members, then piloted the Virtual Village for one month (August–September, 2022). This was done with approval from the University of California, Riverside Institutional Review Board. As a part of the registration and vetting process, participants disclosed their HIV status to the research team. Training sessions detailing how to navigate Discord were offered to participants.

As detailed in Figure 1, we embedded six interventions into the Virtual Village during the pilot period. These included social mixers, a buddy system, expert presentations, resources, guided discussions and mindful meditation. Interventions were selected based on recommendations from CAB members.

**Figure 1:**
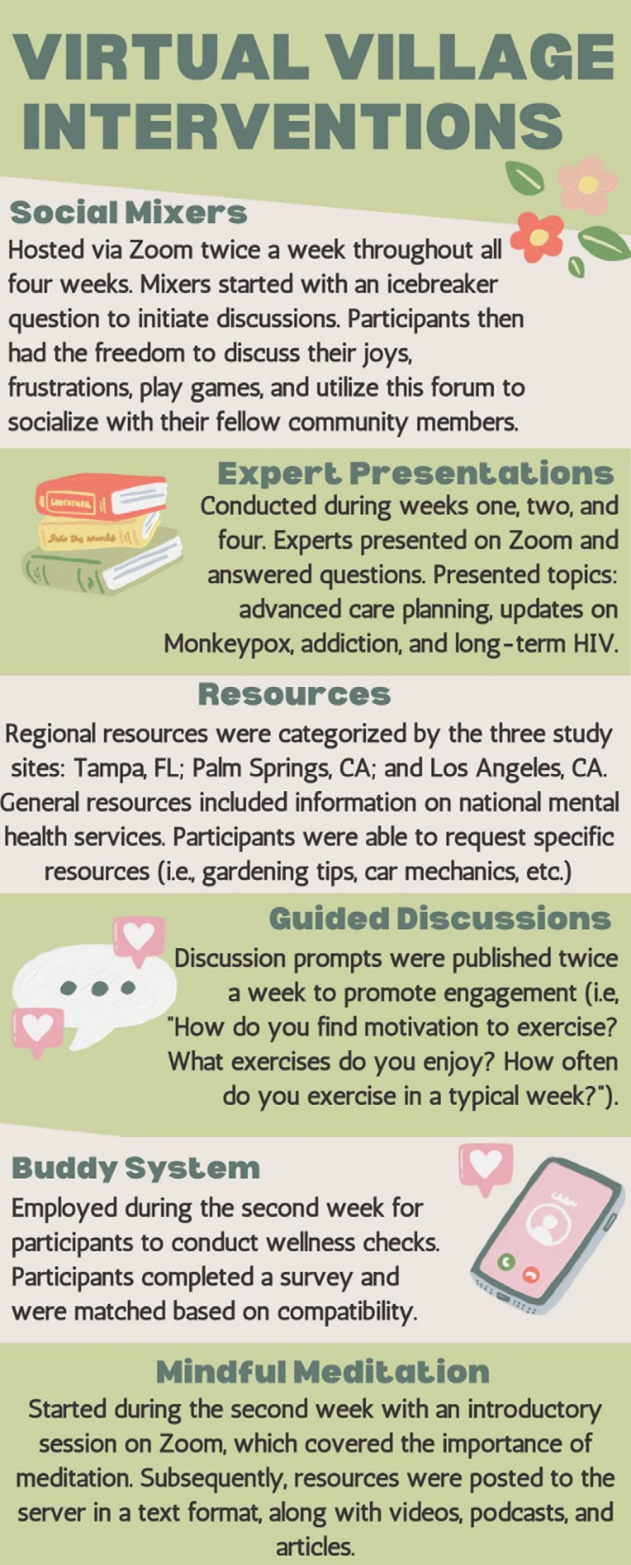
Descriptions of the six interventions implemented during the month‐long pilot.

Research team members moderated the Virtual Village pilot, observed participant interactions and encouraged participant feedback. Presentations were provided by topical experts, and resource pages were created by the CAB. Pilot participants modified these pages to ensure that peers had access to trusted information and services.

Social mixers were hosted biweekly. Frequent attendees participated in mixers at least weekly, allowing them to maintain social connections with their peers. Other social activities included a buddy system and guided discussions. The buddy system was created for OPLH to build closer relationships with those with similar interests, and a buddy match reveal ceremony was initiated to facilitate engagement. Several participants expressed their satisfaction with their pairings and enjoyed meeting with their matches outside of the Virtual Village. Guided discussions were also popular with participants; initially, discussion prompts were posted once per week but were increased to biweekly upon participants’ request. Additionally, subcommunities were developed and added to the server as “channels” based on participants’ shared interests (e.g. gardening, pets, arts and crafts).

Of the 24 Virtual Village pilot participants, 20 (83%) completed both pre‐ and post‐surveys, which included questions about participation experiences. Participants were 51–77 years old (mean = 63). Most identified as male (*n* = 15) and gay (*n* = 13). When asked an open‐ended question as to how the Virtual Village could be improved, common responses included requests to improve the overall interface, use a more user‐friendly platform, give more advanced notice about events and simplify the platform onboarding process. Some individuals also reported that they would enjoy more social events, suggesting that social activities fuelled participants to engage with one another and the platform. When asked to rate their satisfaction interacting with research staff on the server (5‐point scale; “very satisfied” to “very unsatisfied”) all were at least “moderately satisfied” and 70% (*n* = 14) indicated they were “very satisfied.” Lastly, when asked if they wanted to continue using the Virtual Village for non‐research purposes after the study ended, 75% (*n* = 15) indicated a positive response. Based on these responses and observations of community engagement, we identified three key lessons:

1. *When working with older populations, be mindful that some may experience significant difficulty using newer technology*. Technological issues associated with the onboarding process for Discord were a primary barrier to participation in the Virtual Village. While our CAB provided crucial insight to the research team in the planning phase of this pilot, greater integration of CAB members into the onboarding and implementation phases could help better anticipate and troubleshoot issues that are common for older people when encountering new technology. To mitigate onboarding issues in future studies, we recommend hosting additional platform training sessions prior to the start of the study to allow for an adjustment period. Other media platforms with innovative and user‐friendly functions should be explored for real‐world scaling up of Virtual Village projects.
2. *Interactive social events were frequently attended and requested by participants*. Future studies focused on community building should consider that participants may initially seek entertaining activities (e.g. social mixers, subcommunities and the buddy system) over didactic lectures, as this allows them to actively build positive social connections.
3. *Living with HIV is not enough to bond individuals*. A unique component of the Virtual Village is the vetting process, which aims to ensure comfort among users in the event they wish to discuss their experiences living with HIV. Such open discussion of shared life experiences could not be accomplished by joining a non‐HIV‐specific platform. Nonetheless, participants noted during social events that living with HIV does not dictate their life. Their diverse personal interests, such as art, pets and gardening, trumped HIV‐specific discussions. Hence, the Virtual Village can be a space to bring OPLH together to discuss shared interests beyond HIV.

## COMPETING INTERESTS

All authors declare no competing interests related to this work.

## AUTHORS’ CONTRIBUTIONS

BJB oversaw the designing process and JLL was responsible for drafting the manuscript. ANP, ALN, KYG, JTG, MYJ and JT provided input. BJB, ANP and JLL collaborated to review the final draft, which was approved by all of the authors.

## FUNDING

Supported in part by research grant 60304 from the Merck Investigator Initiated Studies Program of Merck & Co, Inc. ALN additionally received support from the NIH/NIA (K01 AG064986).

## DISCLAIMER

The opinions expressed in this paper are those of the authors and do not necessarily represent those of Merck & Co, Inc. or the US National Institutes of Health.

## Data Availability

The data that support the findings of this study are available from the corresponding author upon reasonable request.
